# Estimation of pulse pressure variation and cardiac output in patients having major abdominal surgery: a comparison between a mobile application for snapshot pulse wave analysis and invasive pulse wave analysis

**DOI:** 10.1007/s10877-020-00572-1

**Published:** 2020-08-04

**Authors:** Phillip Hoppe, Fabian Gleibs, Luisa Briesenick, Alexandre Joosten, Bernd Saugel

**Affiliations:** 1 Department of Anesthesiology, Center of Anesthesiology and Intensive Care Medicine, University Medical Center Hamburg-Eppendorf, Martinistrasse 52, 20246 Hamburg, Germany; 2 Department of Anesthesiology, CUB Erasme University Hospital, Université Libre de Bruxelles, 808 Route de Lennik, 1070 Brussels, Belgium; 3 Department of Anesthesiology and Intensive Care, Hôpitaux Universitaires Paris-Sud, Université Paris-Sud, Université Paris-Saclay, Hôpital De Bicêtre, Assistance Publique Hôpitaux de Paris (AP-HP), Le Kremlin-Bicêtre, France; 4 Outcomes Research Consortium, Cleveland, OH USA

**Keywords:** Non-invasive, Hemodynamic monitoring, Cardiovascular dynamics, Fluid management, Fluid responsiveness, Blood flow

## Abstract

Pulse pressure variation (PPV) and cardiac output (CO) can guide perioperative fluid management. Capstesia (Galenic App, Vitoria-Gasteiz, Spain) is a mobile application for snapshot pulse wave analysis (PWAsnap) and estimates PPV and CO using pulse wave analysis of a snapshot of the arterial blood pressure waveform displayed on any patient monitor. We evaluated the PPV and CO measurement performance of PWAsnap in adults having major abdominal surgery. In a prospective study, we simultaneously measured PPV and CO using PWAsnap installed on a tablet computer (PPV_PWAsnap_, CO_PWAsnap_) and using invasive internally calibrated pulse wave analysis (ProAQT; Pulsion Medical Systems, Feldkirchen, Germany; PPV_ProAQT_, CO_ProAQT_). We determined the diagnostic accuracy of PPV_PWAsnap_ in comparison to PPV_ProAQT_ according to three predefined PPV categories and by computing Cohen’s kappa coefficient. We compared CO_ProAQT_ and CO_PWAsnap_ using Bland-Altman analysis, the percentage error, and four quadrant plot/concordance rate analysis to determine trending ability. We analyzed 190 paired PPV and CO measurements from 38 patients. The overall diagnostic agreement between PPV_PWAsnap_ and PPV_ProAQT_ across the three predefined PPV categories was 64.7% with a Cohen’s kappa coefficient of 0.45. The mean (± standard deviation) of the differences between CO_PWAsnap_ and CO_ProAQT_ was 0.6 ± 1.3 L min^− 1^ (95% limits of agreement 3.1 to − 1.9 L min^− 1^) with a percentage error of 48.7% and a concordance rate of 45.1%. In adults having major abdominal surgery, PPV_PWAsnap_ moderately agrees with PPV_ProAQT_. The absolute and trending agreement between CO_PWAsnap_ with CO_ProAQT_ is poor. Technical improvements are needed before PWAsnap can be recommended for hemodynamic monitoring.

## Introduction

The assessment of fluid responsiveness using dynamic cardiac preload variables such as pulse pressure variation (PPV) and the estimation of cardiac output (CO) are mainstays of perioperative fluid management [1–3]. PPV and CO can be measured using pulse wave analysis of the arterial blood pressure waveform [4–6]. Because this technology is not included in most routine patient monitors, hemodynamic monitoring using pulse wave analysis requires advanced hemodynamic monitors. This may limit the clinical use of pulse wave analysis, especially in low-resource settings.

A promising approach to overcome the problem of additional hemodynamic monitoring equipment is the development of innovative mobile monitoring techniques using smartphones and tablet computers [7, 8]. The Capstesia application (Galenic App, Vitoria-Gasteiz, Spain), that can be installed on smartphones or tablet computers, is a mobile application for snapshot pulse wave analysis (PWAsnap). It was proposed to estimate PPV and CO by pulse wave analysis of a snapshot of the arterial blood pressure waveform displayed on any patient monitor screen. In-silico proof of concept studies demonstrated that PWAsnap is basically able to estimate PPV and CO from a waveform snapshot [9, 10]. However, the few clinical method comparison studies evaluating PWAsnap used different PPV and CO reference methods and revealed inconsistent results [11–15].

Before PWAsnap can be considered for routine hemodynamic monitoring its measurement performance needs to be investigated in comparison to established reference methods. We, therefore, performed a prospective study to compare PPV and CO estimated by PWAsnap with PPV and CO determined using invasive internally calibrated pulse wave analysis in adults having major abdominal surgery.

## Materials and methods

### Study design and setting

This prospective method comparison study was approved by the ethics committee (ethics committee approval number: PV5825, Ethikkommission der Ärztekammer Hamburg, Hamburg, Germany). All participants gave written informed consent. This study was performed at the University Medical Center Hamburg-Eppendorf (Hamburg, Germany) between November 2018 and October 2019.

### Inclusion and exclusion criteria

Consenting patients > 18 years were eligible for study inclusion if they were scheduled for open major abdominal surgery (radical cystectomy, pancreaticoduodenectomy, ovarian cancer surgery, and partial hepatectomy) and when advanced hemodynamic monitoring using invasive internally calibrated pulse wave analysis with an arterial catheter was planned independently from the study. Exclusion criteria were a history or presence of atrial fibrillation or excessive premature atrial or ventricular contractions.

### Anesthetic management

General anesthesia was induced using sufentanil, propofol, and a muscle relaxant (usually rocuronium). General anesthesia was maintained either with propofol or inhaled sevoflurane and sufentanil boluses. In addition to routine anesthetic monitoring, arterial blood pressure was continuously recorded using a 20 g radial arterial catheter. The arterial blood pressure waveform was displayed on the patient monitor routinely used in our institution (Infinity Delta monitor; Dräger Medical Deutschland, Lübeck, Germany). The tidal volume was set to 8 mL kg^− 1^ predicted body weight during study measurements. Predicted bodyweight was calculated as 50 + 0.91 × (height [cm] − 152.4) for male and as 45.5 + 0.91 × (height [cm] − 152.4) for female patients [16].

### Study measurements and data extraction

We used PWAsnap to estimate PPV (PPV_PWAsnap_) and CO (CO_PWAsnap_). For this, the Capstesia application was installed on a SPC GLOW 10.1 tablet computer with an integrated camera (Smart Products Connection S.A., Miñano, Álava, Spain). We set the sweep speed of the Infinity Delta monitor to 12.5 mm s^−^^1^ to display at least six to eight cardiac cycles on the monitor. The Infinity Delta monitor always displays a dotted line at half of the scale. This dotted line cannot be deactivated and interferes with the PWAsnap measurement. By setting the scale to the maximum of 300 mmHg, we ensured that the dotted line on the screen did not cross the arterial blood pressure waveform displayed on the screen. By adjusting sweep speed and scale, we optimized conditions for using PWAsnap with the Infinity Delta monitor.

We took snapshots of the arterial blood pressure waveform with the integrated two megapixels camera of the tablet computer. To take a snapshot, the tablet computer had to be held parallel to the patient monitor. The snapshot was cropped to display the arterial blood pressure waveform only. Heart rate as well as systolic and diastolic arterial blood pressure were manually entered afterwards as required by PWAsnap.

We used the ProAQT system (Pulsion Medical Systems, Feldkirchen, Germany) to measure PPV (PPV_ProAQT_) and CO (CO_ProAQT_) using invasive internally calibrated pulse wave analysis of the arterial blood pressure waveform. The sensor of the ProAQT system was put in series to the standard pressure transducer connected to the Infinity Delta monitor. The ProAQT system estimates CO based on features of the arterial blood pressure waveform and biometric data without any external calibration.

We simultaneously measured PPV_PWAsnap_ and PPV_ProAQT_ as well as CO_PWAsnap_ and CO_ProAQT_ during surgery at five time points with at least five minutes between two measurements. To obtain simultaneous measurements, we recorded PPV_ProAQT_ and CO_ProAQT_ at the moment when a snapshot with PWAsnap was taken. Before each measurement, we zeroed the pressure transducer connected to the Infinity Delta monitor and ProAQT system. Measurements were performed during steady state hemodynamic conditions (i.e., no changes in vasoactive agents or anesthetic management).

### Statistical analysis

We present continuous data as mean ± standard deviation (SD) and categorical data as absolute values (n) with relative frequencies in percent. Linear regression analysis of CO values was performed and illustrated in a scatter plot. We performed Bland-Altman analysis for multiple observations per individual [17]. For Bland-Altman analysis, we subtracted PPV_PWAsnap_ from PPV_ProAQT_ and CO_PWAsnap_ from CO_ProAQT_ and calculated the mean of the differences with the accompanying upper and lower 95% limits of agreement (95%-LOA; mean of the differences ± 1.96 × standard deviation of the mean of the differences). As described previously [12], the diagnostic accuracy of the PPV measurements was determined by categorizing the PPV values in three predefined categories reflecting clinical decision making (PPV < 9%, gray zone PPV 9–13%, PPV > 13%) [12, 18]. For the assessment of the diagnostic accuracy the agreement between PPV_PWAsnap_ and PPV_ProAQT_ across the three predefined PPV categories was calculated. Furthermore, Cohen’s kappa was computed to additionally evaluate the diagnostic accuracy [19]. For CO, we calculated the percentage error between the two methods as 1.96 × standard deviation of the mean of the differences divided by the mean CO of both methods. The percentage error threshold for clinical interchangeability was a priori set at 30% as described previously [20]. For CO, we additionally performed four-quadrant plot analysis to evaluate the trending ability [21]. For four-quadrant plot analysis, we plotted the difference of consecutive CO_PWAsnap_ values (ΔCO_PWAsnap_) on the y-axis and difference of consecutive CO_ProAQT_ values (ΔCO_ProAQT_) on the x-axis. A central exclusion zone of 0.5 L min^− 1^ was applied to exclude clinically unimportant small changes. The concordance rate is the ratio (in percent) of the ΔCO values that change in the same direction in relation to all ΔCO values [21]. A change in the same direction means that both ΔCO values either increased or decreased. For statistical analysis we used MedCalc Version 19.1.3 (MedCalc Software, Ostend, Belgium), IBM SPSS Version 25 (IBM, Armonk, NY, USA), and GraphPad PRISM (GraphPad Software, San Diego, USA).

## Results

We included a total of 50 patients. Surgery was cancelled in two patients. We excluded one patient because of new-onset atrial fibrillation. Two patients were excluded because the patients were monitored with a monitoring system other than the ProAQT system. We additionally had to exclude seven patients due to unavoidable overlap between the arterial blood pressure waveform and the dotted line on the screen of the Infinity Delta monitor. This made measurements using PWAsnap impossible. We thus analyzed data of 38 patients with 190 paired PPV and CO measurements. Table 1 shows patient characteristics and intraoperative data.

**Table 1:** Patient characteristics

Demographic and biometric data	
Male sex, [n (%)]	11 (29)
Age, mean ± SD [years]	64 ± 11
Height, mean ± SD [cm]	168 ± 10
Weight, mean ± SD [kg]	78 ± 20
Predicted body weight, mean ± SD [kg]	61 ± 11
Body Mass Index, mean ± SD [kg m^− 2^]	28 ± 7
Type of surgery	
Radical cystectomy, [n (%)]	12 (31.6)
Pancreaticoduodenectomy, [n (%)]	13 (34.2)
Ovarian cancer surgery, [n (%)]	12 (31.6)
Partial hepatectomy, [n (%)]	1 (2.6)

Data are shown as mean ± standard deviation (SD) or absolute (n) and relative frequencies (%)

**Fig. 1:**
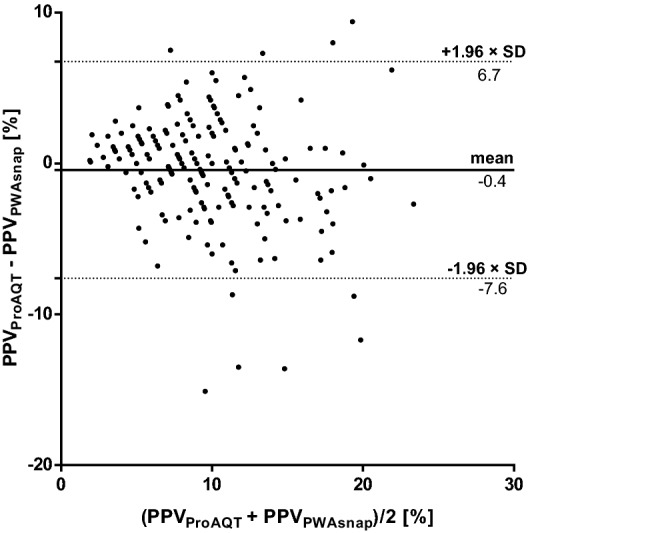
Bland-Altman plot comparing pulse pressure variation (PPV) measured using mobile application pulse wave analysis (PWAsnap) and invasive pulse wave analysis. The bold line represents the mean of the differences between PPV measured using the two methods. The dotted lines represent the 95%-limits of agreement. *PPV*_*PWAsnap*_ pulse pressure variation determined with PWAsnap, *PPV*_*ProAQT*_ pulse pressure variation determined with ProAQT system

Mean PPV_PWAsnap_ was 10.1 ± 5.1% and mean PPV_ProAQT_ was 9.7 ± 4.4%. The mean of the differences between PPV_PWAsnap_ and PPV_ProAQT_ was − 0.4 ± 3.7% (95%-LOA 6.7 to − 7.6%) (Fig. 1). The distribution of PPV_PWAsnap_ and PPV_ProAQT_ across the three predefined PPV categories is shown in Table 2. The overall diagnostic agreement between PPV_PWAsnap_ and PPV_ProAQT_ across the three predefined PPV categories was 64.7% with a Cohen’s kappa coefficient of 0.45.

**Table 2:** Distribution and diagnostic agreement of pulse pressure variation measurements across the three predefined categories

	PPV_PWAsnap_			Total
PPV _ProAQT_	< 9%	9–13%	> 13%	
< 9%	**63 (72%)**	17 (20%)	*7 (8%)*	87 (100%)
9–13%	23 (33%)	**33 (48%)**	13 (19%)	69 (100%)
> 13%	*0 (0%)*	7 (21%)	**27 (79%)**	34 (100%)

*PPV*_*PWAsnap*_ pulse pressure variation measured with mobile application pulse wave analysis, *PPV*_*ProAQT*_ pulse pressure variation measured with ProAQT system, *bold* measurement pairs in concordant category, *italic* measurement pairs in opposite category

Percentages are calculated for each horizontal row

**Fig. 2:**
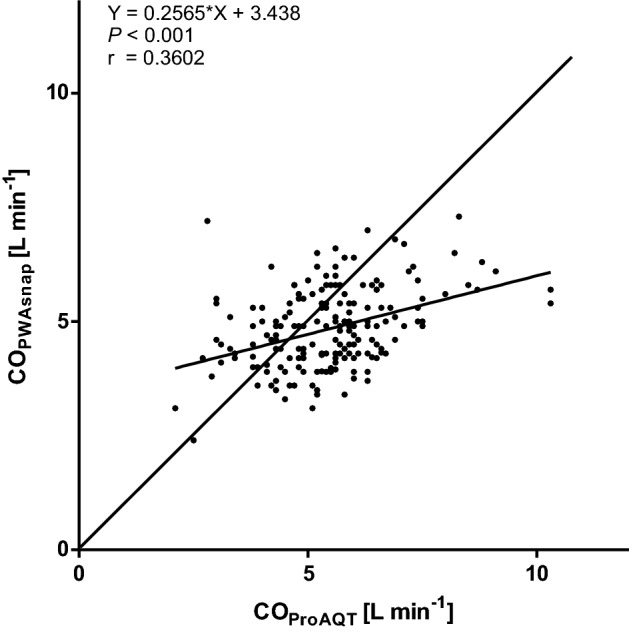
Scatter plot with linear regression analysis of cardiac output measured using mobile application pulse wave analysis (PWAsnap) and invasive pulse wave analysis. *CO*_*PWAsnap*_ cardiac output estimated with PWAsnap, *CO*_*ProAQT*_ cardiac output estimated with ProAQT system, *Y* slope-intercept equation, *r* correlation coefficient

**Fig. 3:**
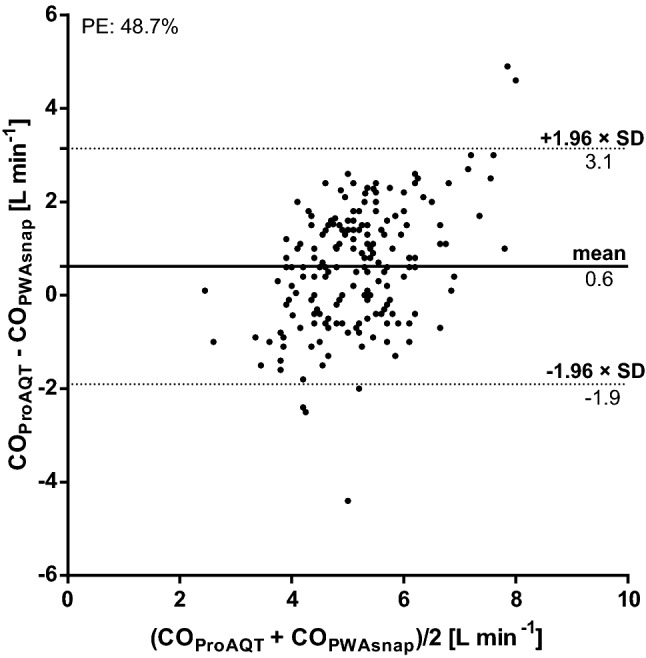
Bland-Altman plot comparing cardiac output (CO) measured using mobile application pulse wave analysis (PWAsnap) and invasive pulse wave analysis. The bold line represents the mean of the differences between CO measured using the two methods. The dotted lines represent the 95%-limits of agreement. *CO*_*PWAsnap*_ cardiac output estimated with PWAsnap, *CO*_*ProAQT*_ cardiac output estimated with ProAQT system, *PE* percentage error

**Fig. 4:**
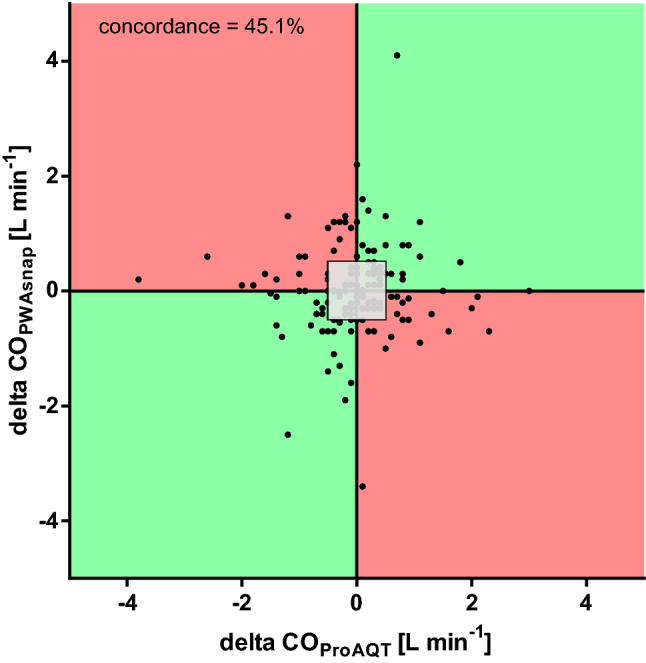
Four-quadrant plot to estimate the concordance rate between cardiac output measured using mobile application pulse wave analysis (PWAsnap) and invasive pulse wave analysis. The gray square is the central exclusion zone of 0.5 L min ^− 1^. *CO*_*PWAsnap*_ cardiac output estimated with PWAsnap, *CO*_*ProAQT*_ cardiac output estimated with ProAQT system

Mean ± SD CO_PWAsnap_ was 4.8 ± 0.9 L min^− 1^ and mean CO_ProAQT_ was 5.5 ± 1.3 L min ^−1^. The relation between CO_PWAsnap_ and CO_ProAQT_ is shown in Fig. 2. The mean of the differences between CO_PWAsnap_ and CO_ProAQT_ was 0.6 ± 1.3 L min^− 1^ (95%-LOA 3.1 to − 1.9 L min^− 1^) (Fig. 3). The percentage error was 48.7%. The concordance rate between changes in CO_PWAsnap_ and CO_ProAQT_ was 45.1% (Fig. 4).

## Discussion

We performed a prospective method comparison study to compare PPV and CO estimated by PWAsnap with PPV and CO determined using invasive internally calibrated pulse wave analysis in adults having major abdominal surgery. The overall diagnostic agreement between PPV_PWAsnap_ and PPV_ProAQT_ across the three predefined PPV categories was moderate according to Cohen’s kappa [19]. The absolute agreement of CO_PWAsnap_ with CO_ProAQT_ was poor and the measurements were not interchangeable. Trending ability of CO_PWAsnap_ was also poor with a low concordance rate.

An in-silico proof of concept study showed that calculating PPV and CO by analyzing snapshots of an arterial blood pressure waveform displayed on a patient monitor is basically possible using PWAsnap [10]. The analysis of these snapshots showed a good concordance with PPV and CO measurements of the raw data [10]. The measurement performance and diagnostic accuracy of PPV_PWAsnap_ has been investigated in previous studies. An experimental study in a highly controlled simulated environment suggested that PPV_PWAsnap_ may be a good substitute for manual PPV determination [9]. Our results on the diagnostic accuracy of PPV_PWAsnap_ are similar to a recent study comparing PPV_PWAsnap_ with stroke volume variation (SVV) determined using the FloTrac system (Vigileo monitor; Edwards Lifesciences, Irvine, CA, USA) in 40 patients having major abdominal surgery [12]. Like in this previous study, we used three predefined PPV categories reflecting clinical decision making according to the “gray zone” approach [18]. When comparing different methods to measure PPV it is more important to know if PPV values fall into the same category and thus result in the same treatment decisions regarding fluid therapy than if PPV values have the exact same numeric value. In contrast to the previous study, we compared PPV_PWAsnap_ directly to PPV_ProAQT_ and not to SVV. Furthermore, the conditions to use PPV were optimized in our study (e.g., sinus rhythm and controlled mechanical ventilation) [16]. In our study, Cohen’s kappa [19] suggested moderate diagnostic agreement between PPV_PWAsnap_ and PPV_ProAQT_ across the three predefined PPV categories in accordance with the previous study [12], but overall agreement appeared to be lower in our analysis. Notably, nearly 80% of the patients with a PPV_ProAQT_>13% were correctly classified by PWAsnap.

The few clinical method comparison studies evaluating CO_PWAsnap_ revealed inconsistent results. CO_PWAsnap_ was compared to CO determined using invasive internally calibrated pulse wave analysis (FloTrac system) in 53 patients during major oncological surgery [14]. In this study, a percentage error of 26% suggested good agreement between CO_PWAsnap_ and FloTrac-derived CO [14]. These findings are in contrast to our study revealing wide 95%-LOA when comparing CO_PWAsnap_ with CO_ProAQT_. Furthermore, the percentage error in our analysis exceeded the threshold for clinical interchangeability of 30% [20]. In another validation study, CO_PWAsnap_ was compared to CO measured with transpulmonary thermodilution, a clinical reference method [22, 23], in 57 patients during cardiac surgery [11]. With a mean of the differences between CO measurements obtained by the two methods of 0.3 L min^− ^^1^, wide 95%-LOA of 3.3 to -2.8 L min^− 1^, a high percentage error of 60%, and a poor concordance rate the results of this study are consistent with our findings [11].

Previously, it was reported that close to 10% of the snapshots of the arterial blood pressure waveform cannot be analyzed by PWAsnap [11]. We did not systematically analyze the quality of the snapshots or the failure rate of PWAsnap to estimate PPV and CO. We thus can only subjectively describe our experiences of using PWAsnap in a clinical setting in the operating room. PWAsnap sometimes failed to analyze a snapshot without an apparent reason. We speculate that the poor camera performance of the tablet computer may have contributed to these difficulties in the analysis of the snapshots. Other studies that used PWAsnap installed on smartphones with high resolution cameras did not report these problems [11, 14].

Considering the results of our study, technical refinements are needed before PWAsnap can be recommended as an alternative to current monitoring methods. In general, using mobile devices in combination with hemodynamic monitoring applications instead of bulky and costly monitoring equipment is a promising approach to future hemodynamic monitoring in the operating room. Most medical personnel use smartphones making such applications almost universally available.

There are limitations of this study. We did not perform interventions such as fluid challenge or passive leg raising tests and, therefore, cannot describe the ability of PPV_PWAsnap_ to actually predict fluid responsiveness. We compared CO_PWAsnap_ with CO_ProAQT_. Invasive internally calibrated pulse wave analysis (ProAQT system) is widely used to guide intraoperative hemodynamic therapy but is not a clinical reference method for CO measurement [22, 23]. However, the ProAQT system exhibits reasonable CO trending ability [24] and a perioperative goal-directed therapy treatment algorithm based on hemodynamic variables obtained with the ProAQT system has been shown to improve patient outcome [25]. We only included patients having major abdominal surgery. Therefore, our results are not necessarily transferable to other clinical situations in the operating room or in the intensive care unit. We did not perform study measurements at predefined time points during surgery (e.g., pre-induction, post-induction, pre-incision etc.), but all measurements were performed post-incision and with at least five minutes between two measurements.

In contrast to other studies evaluating PWAsnap [9, 11, 12], we used singular PWAsnap measurements and did not calculate a mean of three or more consecutive measurements. We think, that this approach reflects how PWAsnap would be used in clinical practice as calculating a mean of multiple measurements may be too complicated and time-consuming.

It has to be noted that PWAsnap is not commercially available at the moment and to the best of our knowledge it is unclear if a new version will be released. Nevertheless, PWAsnap remains a highly innovative approach to hemodynamic monitoring and our study comparing PWAsnap with an established reference method may provide information that is helpful for the development of future versions or new technologies for PPV and CO monitoring based on PWAsnap.

In conclusion, in adults having major abdominal surgery, PPV_PWAsnap_ moderately agrees with PPV_ProAQT_ and the absolute and trending agreement between CO_PWAsnap_ with CO_ProAQT_ is poor. Technical improvements are needed before PWAsnap can be recommended as an alternative to current monitoring methods.

## Data Availability

Data are available from the authors upon reasonable request.
